# Investigating Host Microbiota Relationships Through Functional Metagenomics

**DOI:** 10.3389/fmicb.2019.01286

**Published:** 2019-06-07

**Authors:** Elisabeth Laville, Josette Perrier, Nada Bejar, Marc Maresca, Jeremy Esque, Alexandra S. Tauzin, Emna Bouhajja, Marion Leclerc, Elodie Drula, Bernard Henrissat, Stephane Berdah, Eric Di Pasquale, Patrick Robe, Gabrielle Potocki-Veronese

**Affiliations:** ^1^INSA, INRA, CNRS, LISBP, Université de Toulouse, Toulouse, France; ^2^iSm2, Centrale Marseille, CNRS, Aix-Marseille University, Marseille, France; ^3^UMR1319, Micalis, Institut National de la Recherche Agronomique, Jouy-en-Josas, France; ^4^CNRS, Architecture et Fonction des Macromolécules Biologiques, Aix-Marseille Université, Marseille, France; ^5^USC 1408 AFMB, INRA, Marseille, France; ^6^UMRT24 IFSTTAR, Laboratoire de Biomécanique Appliquée, Faculté de Médecine Secteur Nord, Aix-Marseille University, Marseille, France; ^7^Inst Neurophysiopathol, INP, CNRS, Aix-Marseille Université, Marseille, France; ^8^Givaudan SA, Toulouse, France

**Keywords:** functional metagenomics, carbohydrate-active enzymes, human intestinal mucin, human gut microbiota, lectin binding

## Abstract

The human Intestinal mucus is formed by glycoproteins, the O- and N-linked glycans which constitute a crucial source of carbon for commensal gut bacteria, especially when deprived of dietary glycans of plant origin. In recent years, a dozen carbohydrate-active enzymes from cultivated mucin degraders have been characterized. But yet, considering the fact that uncultured species predominate in the human gut microbiota, these biochemical data are far from exhaustive. In this study, we used functional metagenomics to identify new metabolic pathways in uncultured bacteria involved in harvesting mucin glycans. First, we performed a high-throughput screening of a fosmid metagenomic library constructed from the ileum mucosa microbiota using chromogenic substrates. The screening resulted in the isolation of 124 clones producing activities crucial in the degradation of human O- and N-glycans, namely sialidases, β-D-N-acetyl-glucosaminidase, β-D-N-acetyl-galactosaminidase, and/or β-D-mannosidase. Thirteen of these clones were selected based on their diversified functional profiles and were further analyzed on a secondary screening. This step consisted of lectin binding assays to demonstrate the ability of the clones to degrade human intestinal mucus. In total, the structural modification of several mucin motifs, sialylated mucin ones in particular, was evidenced for nine clones. Sequencing their metagenomic loci highlighted complex catabolic pathways involving the complementary functions of glycan sensing, transport, hydrolysis, deacetylation, and deamination, which were sometimes associated with amino acid metabolism machinery. These loci are assigned to several *Bacteroides* and *Feacalibacterium* species highly prevalent and abundant in the gut microbiome and explain the metabolic flexibility of gut bacteria feeding both on dietary and human glycans.

## Introduction

The human intestinal tract harbors a dense commensal microbial community whose interactions with the host are of paramount importance for its health and well-being, particularly for the development of immunity and protection against invasion by pathogens. One of the best-known functions of the microbiota is its contribution to the digestion of dietary fibers, derived mainly from plant cell walls. Dietary fibers, consisting of polysaccharides of great structural diversity, represent the main energy source for the microbiota. The majority of gut bacterial species possess an enzymatic arsenal in the form of carbohydrate-active enzymes (CAZymes; Lombard et al., 2014) used to depolymerize these fibers. The products are further fermented to provide Short chain fatty acids (SCFAs) to the host. By contrast CAZymes produced by the human digestive system can only act on digestible starch and some other simple sugars such as sucrose or lactose (El Kaoutari et al., 2013). In addition to dietary fibers, intestinal microorganisms use glycans associated with human glycoproteins as a source of energy in particular mucin glycans that are secreted by the goblet cells of the intestinal epithelium. Mucin glycoproteins contain a large panel of mostly O-linked, and some N-linked, glycan structures (Podolsky, 1985; Robbe et al., 2004). Mucins represent a protective barrier for the intestine that is perpetually renewed. Mucus-colonizing bacteria produce both proteins to adhere to mucins and enzymes to degrade them (Tailford et al., 2015). The enzymatic machinery of these bacteria allows mucins to be recycled.

In recent years, growth assays on pig and human mucins, combined with pioneering genomic and transcriptomic studies, have shown that a number of pathogens and commensals (Tailford et al., 2015; Martens et al., 2018), including several *Bacteroides* species (Bäckhed et al., 2005; Sonnenburg et al., 2005; Martens et al., 2008; Marcobal et al., 2013), *Ruminococcus gnavus* (Owen et al., 2017) and the probiotic species *Akkermansia muciniphila* (Derrien et al., 2004), are involved in mucin degradation. This was especially described in cases of dietary fiber deprivation, when certain commensals shift their metabolism from dietary to host glycans (Desai et al., 2016). Furthermore, recent studies have also shown correlations between host diet, a reduction in mucus thickness, microbiota composition, and inflammatory bowel diseases (ulcerative colitis and Crohn’s disease) and colorectal cancers (Pickard et al., 2014; Jakobsson et al., 2015; Desai et al., 2016). Despite these recent advances, only a dozen CAZymes (reviewed in Tailford et al., 2015) have been biochemically proven to degrade mucin oligosaccharides, and our understanding of mucin glycan-degrading pathways is almost exclusively restricted to cultured species. To date, only one enzyme has been characterized as involved in the degradation of human N-glycans by an uncultured gut bacterium (Ladevèze et al., 2013, 2015). Considering the fact that the majority of microbiota species are still uncultured, the studies so far conducted into the degradation of human glycans by gut bacteria are thus far from exhaustive.

In this study, we used functional metagenomics to identify mucin-degrading pathways from the uncultured fraction of the human gut microbiota. A two-step activity-based screening strategy was applied to search for mucin catabolic pathways in a mucosal ileal metagenomic library, and to demonstrate their involvement in the structural modification of human colon mucus. Genomic and metagenomic data were integrated to assess the abundance and prevalence of these mucin glycan utilization loci in the human gut microbiome, and to reveal synteny with genomic loci from reference gut bacteria, which had not previously been proven to be involved in these host–microbiota relationships.

## Materials and Methods

### Metagenomic DNA Sampling and Library Construction

Bacterial metagenomic DNA was obtained from the distal ileum sample of a 50-55-year-old patient undergoing colonoscopy and surgery for a suspected cancer of the lower colon after patient had been subjected to a cleansing preparation. The patient did not receive any antibiotics or other drugs in the 6 months before sampling. The sampling protocol was approved by the local ethics committee: the Comité de Protection des Personnes Sud Est V (Ref.: 07-CHUG-21; B. Habozit, J. L. Crakowski, J. Juge, J. Grunwald, E. Svhan, and E. Fontaine on the committee). A segment of 2 cm^2^ was obtained from a healthy zone, before being immediately frozen and kept at -80°C until processing. The ileal mucosa was scraped, an enriched bacterial fraction was recovered using the method described in Courtois et al. (2003), and the metagenomic DNA was extracted as described in Tasse et al. (2010). Fragments of between 30 and 40 kb in size were isolated and cloned into the pCC1FOS fosmid (Epicenter Technologies). EPI100 *E. coli* cells were then transfected to obtain a library of 20,000 clones. Recombinant clones were transferred to 384-well microtiter plates containing a Luria-Bertani (LB) medium, supplemented with 12.5 mg/L chloramphenicol and 8% (w/v) glycerol. They were grown for a period of 22 h at 37°C and then frozen and conserved at -80°C. All other culture media mentioned in this study contained 12.5 mg/L chloramphenicol.

### Metagenomic Library Screening

The library was gridded using an automated microplate gridder (K2, KBiosystem, Basildon, United Kingdom) on 22 cm × 22 cm trays containing a solid agar medium supplemented with 12.5 mg/L chloramphenicol and with chromogenic substrates at a final concentration of 60 μg/mL (w/v). The chromogenic substrates used – 5-Bromo-4-chloro-3-indolyl a-L-fucopyranoside (X-α-L-Fuc), 5-Bromo-4-chloro-3-indolyl 2-acetamido-2-deoxy-b-D-glucopyranoside (X-β-D-GlcNac), 5-Bromo-4-chloro-3-indolyl N-acetyl-a-D-neuraminic acid (X-α-D-Neu5Ac), 5-Bromo-4-chloro-3-indolyl 2-acetamido-2-deoxy-b-D-galactopyranoside (X-β-D-GalNac), 5-Bromo-4-chloro-3-indolyl a-D-mannopyranoside (X-α-D-Man), and 5-Bromo-4-chloro-3-indolyl b-D-mannopyranoside (X-β-D-Man) – were purchased from Carbosynth^1^. The plates were incubated at 37°C for periods of between 1 day and 2 weeks, depending on the time needed to observe the blue-colored clones.

### Lectin Binding Experiments

On the basis of their multiple activities on the chromogenic X-substrates from the primary screening, a selection of hit clones were tested for their ability to degrade human colon mucus using lectin binding assays. The hit clones were grown at 37°C in 400 mL LB medium, with orbital shaking at 120 rpm. After 16 h, cells were harvested using centrifugation for 5 min at 5,000 rpm, before being re-suspended and concentrated in an activity buffer (RPMI medium from Sigma) to obtain a final OD600 nm of 80. Cell lysis was carried out using sonication. Cell debris were centrifuged at 13,000 rpm for 10 min and cytoplasmic extracts were filtered using a 0.20 μm Minisart RC4 syringe filter. An *E. coli* EPI100 clone containing the pCC1FOS fosmid without a metagenomic DNA fragment was used as a negative control. The human colon mucus was isolated from three patients of both genders, 31–74 years of age and all of European origin as previously described (Ajandouz et al., 2016). The sampling procedures were approved by the French Ethics Committee (CODECOH no. DC-2011-1319). Samples were taken from a macroscopically unaffected area as identified by the surgeon. After resection, the specimens were placed in an ice-cold DMEM solution supplemented with antibiotics. After longitudinal opening of the intestine, the surface of the mucosa was scraped in PBS to collect intestinal mucus. The mucus from the three patients was pooled and stored at -80°C until used. A lectin binding assay was used to test the effect of bacterial enzymes expressed by the positive clones on human mucins. Briefly, 96-well plates (F-Bottom, Nunc Maxisorp^®^) were coated overnight at 4°C with 100 μL of human mucins (5 μg/mL) diluted in a pH 9.6 bicarbonate/carbonate coating buffer (100 mM). After two washes with 200 μL phosphate saline buffer (pH 7.4) supplemented with Tween20 0.02% (v/v; PBS-T), the plates were incubated at 37°C with 100 μL of the bacterial supernatant (dilution: 1/100) for 5 h. The wells were washed twice with 200 μL PBS-T before being saturated at room temperature for 1 h with 200 μL PBS containing 1% BSA (PBS-B). Finally, the wells were washed twice with 200 μL PBS-Tween, and 100 μL of fluorescein-labeled lectin solution (5 μg/mL) in PBS-B were added. The fluorescein-conjugated lectins used (all from Vector Laboratories, France) were: Concanavalin A (Con A; selective of Manα2/6/3-manβ4-Glc(Nac)-R > α-Man > α-Glc > αGlcNAc), *Sambucus nigra* (soybean) agglutinin (SNA; selective of Neu5Acα1/6 Gal(NAc)-R) and *Triticum vulgaris* (wheat germ) agglutinin (WGA; selective of Galβ4-GlcNacβ6/3-Galβ4-R > GlcNAcβ-R > Neu5Acα3/6/8-R). After 1 h incubation at 37°C, the wells were washed and the fluorescence measured at an excitation/emission of 490 nm using a BioTek Synergy HTX Multi-Mode Microplate Reader (BioTek, France). Each binding experiment for a given lectin was performed in triplicate, using the same microplate for all hit clones including three control clones (*E. coli* host strain transformed with the empty vector Epi100).

The mean fluorescence value for the three control clones, corresponding to 100% binding, was used to calculate the percentage of the lectin binding for each clone, including the three control clones. The variance within biological replicates of each clone was first assessed using the *F*-test against control clones. Subsequently, *p*-values were calculated using the *t*-test to evaluate the statistical significance of differences between a given clone and the controls. *P*-values lower than 0.05 were considered statistically significant.

### Metagenomic Sequence Analysis

The fosmid DNA of the clone hits was extracted using the NucleoBond Xtra Midi kit from Macherey-Nagel (France). Fosmids were then sequenced using the Ion Torrent S5 System at the GeT-Biopuces Platform (Toulouse, France). Read assembly was performed using Masurca^2^. The assembled contigs were cleaned from the pCC1FOS vector sequence using Crossmatch^3^. ORF detection and functional annotation was performed using the RAST annotation server^4^ (Aziz et al., 2008). CAZyme encoding genes were identified by BLAST analysis of the predicted ORFs against the full-length sequences of glycoside hydrolases (GH), polysaccharide lyases (PL), carbohydrate esterases (CE), carbohydrate-binding modules (CBM), and glycosyltransferases (GT) included in the CAZy database^5^, using a cut-off *E*-value of 7⋅10-6. Sequences that aligned over their entire length with a sequence in the database with >50% identity were directly assigned to the same family as the subject sequence. The remaining ones were subjected in parallel to (i) a BLAST search against a library built with partial sequences corresponding to individual GH, PL, CE, CBM, and GT modules and (ii) a HMMER2 search using Hidden Markov models (HMM2) built for each CAZy module family, allowing a view of CAZyme modularity (Lombard et al., 2014). The contigs’ entire nucleotidic sequences were blasted against the non-redundant database of the NCBI and against each other to examine their divergence from the reference strain genomes, and the redundancy between clone hits. Taxonomic assignation of the metagenomic sequences was determined using the PhyloPythias program^6^ (model type: Generic 2013–800 Genera). The same results were obtained with a minimum slice at 3% and 50%. The presence of signal peptide cleavage sites in ORF amino acid sequences were predicted using the SignalP server^7^ (Nielsen, 2017).

### Prevalence Analyses

The homolog sequences of the translated ORFs were searched for in the translated catalog of 9.9 million reference genes using BLASTP (*E*-value = 0, identity ≥ 90%). This catalog consisted of the gut metagenomic sequences of 1,267 subjects from three continents (United States, China and Europe): 139 US HMP samples, 760 European fecal samples from the MetaHIT project and 368 Chinese fecal samples (Li et al., 2014). The microbial gene richness in the human gut was assessed by recovering the occurrence frequency data of homologous sequences of the catalog from the 9.9 million gene frequency table in the 1,267 subjects^8^. The frequency values have no unit. They are normalized to account for sampling and sequencing biases generated by the diverse origins of the cohorts constituting the catalog.

### Data Deposition

The datasets generated in the course of this study are available in the repository of the DDBJ/EMBL/GenBank Nucleotide Sequence Database under accession numbers LR131274-LR131286^9^.

## Results and Discussion

### Screening the Human Gut Metagenome for Mucin Glycan-Degrading Activities

The 20,000 clones of the *E. coli* fosmid library constructed from the ileum mucosa microbiota, covering in total 0.7 Gb of metagenomic sequence, were screened for nearly all the α- and β-glycosidase activities required for the degradation of N- and O-glycans. Firstly, α-L-fucosidases, β-D-N-acetyl-glucosaminidases, β-D-N-acetyl-galactosaminidases, α-D-neuraminic-acid hydrolases, α-D- and β-D-mannosidases were searched for using chromogenic reagents (5-Bromo-4-chloro-3-indoxyl-glycosyl, known as X-glycosyl substrates) in a rich solid medium on which the metagenomic clones had been gridded. It was not possible to screen for β-D-galactosidases using this approach due to the high background activity of the *E. coli* host.

These 120,000 assays allowed 158 validated activities to be identified, with this corresponding to 124 positive clones, since 40 clones produced several of the screened activities (Table 1). Strikingly, no positive clone was found on the fucosyl substrate, despite α-linked-L-fucosyl residues frequently being found at the non-reducing end of O-linked oligosaccharides, and α-fucosidase activity being widespread in gut bacteria whatever their taxa (Katayama et al., 2005; Tailford et al., 2015). This may be due to the fact that α-fucosidases are known to be inactive on this kind of artificial substrate (Katayama et al., 2005). The hit yields varied from 0.1% (for X-α-D-Man) to 3.3% (for X-β-D-GlcNac) depending on the screening substrate, which is on average 2.2 times higher than the hit rate (0.4–1.3%) obtained when screening the same library for dietary fiber hydrolytic activities (Cecchini et al., 2013).

**Table 1:** Results of primary screening.

**Substrates**	**Number of positive clones – Hit rate**
X-β-D-GalNac	47 – 2,3‰
X-β-D-GlcNac	67 – 3,3‰
X-α-D-Man	2 – 0,1‰
X-α-D-Neu5Ac	27 – 1,3‰
X-β-D-Man	17 – 0,8‰
X-α-L-Fuc	0
**Single activities**	**Number of positive clones**
X-β-D-GalNac	15
X-β-D-GlcNac	29
X-α-D-Man	1
X-α-D-Neu5Ac	20
X-β-D-Man	9
**Multiple activities**	**Number of positive clones**
X-β-D-Man + X-α-D-Neu5Ac	2
X-β-D-GlcNac + X-α-D-Neu5Ac	1
X-β-D-GlcNac + X-β-D-Man	2
X-β-D-GlcNac + X-β-D-GalNac	27
X-β-D-GlcNac + X-α-D-Man	1
X-β-D-GlcNac + X-β-D-GalNac + X-β-D-Man	1
X-β-D-GlcNac + X-β-D-GalNac + X-α-D-Neu5Ac	3
X-β-D-GlcNac + X-α-D-Man+ X-β-D-Man	2
X-β-D-GlcNac + X-β-D-GalNac + X-α-D-Neu5Ac + X- β-D-Man	1

### Assessment of Human Intestinal Mucus Degradation

Since chromogenic substrates do not represent the structural complexity of mucin oligosaccharides, we then confirmed the activity of the hit clones on the physiological substrate targeted by mucus-degrading bacteria. From the 124 positive clones, we selected 13 to be tested on human intestinal mucus and/or sequenced. The clones chosen were those: (i) producing complementary glycosidase activities required to break down complex mucin oligosaccharide structures thanks to the expression of several CAZy-encoding genes clustering in the same metagenomic loci, or thanks to the production of a highly promiscuous CAZyme; and (ii) producing the highest levels of activity, defined as those with the quickest response to chromogenic testing.

**FIGURE 1:**
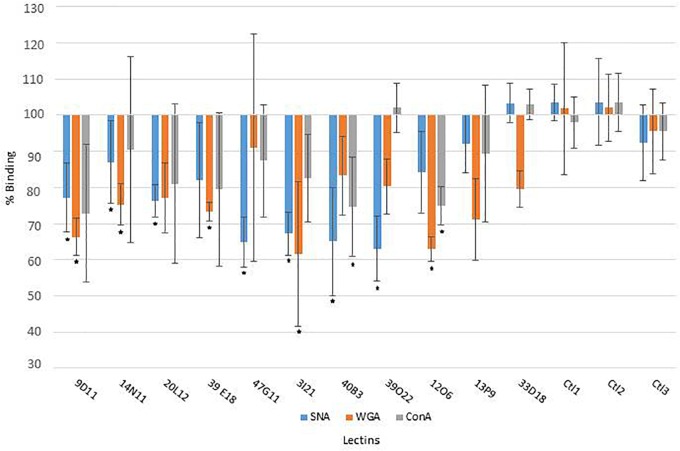
Lectin binding on human colon mucus after incubation with the clone extracts, compared to reference clones (*E. coli* host strain transformed with the empty vector Epi100; ^∗^, significant modification of lectin binding).

The mucus-degrading ability of the metagenomic clones was assessed using the ELISA assay technique, which involves the modification of mucin-specific lectin binding if the epitopes are affected by CAZyme activity. The mucus was incubated with the hit clones’ cytoplasmic extracts. After a washing step, it was then incubated with three fluorescent lectins specific to different N- and O-glycan motifs (Table 2). A comparison of lectin binding percentages with those obtained for reference clones (*E. coli* host strain transformed with the empty vector Epi100) is provided in Figure 1 and Table 2. We observed considerable variability in the binding inhibition values, likely due to the heterogeneousness of the glycan structures in the human mucus samples. Nevertheless, binding modification was significant for several clone–lectin couples. Overall, most of the selected clones, except 13P9 and 33D18, induced a decrease in binding of at least one lectin. The effects were particularly clear for the clones producing activities that target the β-D-GalNAc, β-D-GlcNAc, and α-D-Neu5Ac residues usually found at the terminal extremities of human mucin glycans, which are the most accessible to exo-acting glycosidases. In most cases, the hit clones produced the activities required to degrade the glycan structures targeted by the specific lectins, thus affecting lectin binding.

**Table 2:** Functional profile and CAZyme-encoding gene content of the hit clones.

Clone identifier	CAZy families and their substrate target inferred from the activities listed in the CAZy database								Activity on X- substrates (GH family inferred as responsible for the clone phenotype)				Lectins and their binding specificities (GH family inferred as responsible for the clone phenotype)		
			
													WGA	SNA	ConA
	β-GlcNac	β-GalNac	α-Neu5Ac	β-Gal	α-Gal(Nac)	β-Man	α-Man	α -Fuc	X-β-GlcNac	X-β-GalNac	X-α-Neu5Ac	X-β-Man	Galβ4-GlcNacβ6/3-Galβ4-R > GlcNAcβ-R > Neu5Acα3/6/8-R	Neu5Acα1/6 Gal(NAc)-R	Manα2/6/3-manβ4-Glc(Nac)-R > α-Man > α-Glc >αGlcNAc
9D11				GH1		GH1			X	–	–	X(GH1)	↘(GH1)	↘	–
14N11	GH20	GH20		GH2		GH2	GH92		X(GH20)	X(GH20)	X	X(GH2)	↘(GH2, GH20)	–	–
20L12	GH20	GH20	GH33	GH2	GH27	GH2			X(GH20)	X(GH20)	X(GH33)	–	–	↘(GH33)	–
39E18	GH20	GH20	GH33	GH2	GH27	GH2			X(GH20)	–	–	–	↘(GH2, GH20, GH33)	–	–
47G11	GH20	GH20	GH33	GH2		GH2	GH92	GH29	–	–	X(GH33)	–	–	↘(GH33)	–
3I21	GH20	GH20	GH33					GH29	X(GH20)	X(GH20)	X(GH33)	–	↘(GH20, GH33)	↘(GH33)	–
40B3			GH33						–	–	X(GH33)	–	–	↘(GH33)	↘
41E6	GH3	GH3	GH33						–	–	X(GH33)	–	nt	nt	nt
39O22			GH33						–	–	X(GH33)	–	–	↘(GH33)	–
12O6	GH20	GH20		GH2		GH2			X(GH20)	X(GH20)	–	–	↘(GH2, GH20)	–	↘
47C24	GH20	GH20		GH2		GH2			X(GH20)	X(GH20)	–	–	nt	Nt	nt
13P9				GH2		GH2			–	–	–	X(GH2)	–	–	–
33D18				GH2		GH2			–	–	–	X(GH2)	–	–	–

*X, activity on X-substrates;↘, significant decrease of lectin binding to colon mucus after incubation with the hit clones, compared to reference (E. coli host strain transformed with the empty vector Epi100); nt, non-tested; –, no significant activity/no alteration in binding.*

With respect to the WGA lectin, which is specific to terminal Galβ4-GlcNacβ6/3-Galβ4-R > GlcNAcβ-R, and to a lesser extent, Neu5Acα3/6/8-R motifs, five β-D-N-acetyl-glucosaminidase-producing clones (9D11, 14N11, 39E18, 3I21, and 12O6) induced less lectin binding than was obtained for the reference clones. The terminal Galβ4 residues were likely removed by the *E. coli* β-galactosidase activity, facilitating access to the β-D-N-acetyl-glucosaminidases produced by the hit clones. Binding of the SNA lectin, which is specific to terminal Neu5Acα1/6 Gal(NAc)-R motifs, was reduced after the action of five clones active on X-α-D-Neu5Ac (20L12, 47G11, 3I21, 40B3, and 39O22) and, surprisingly, clone 9D11, which is not active on X-α-D-Neu5Ac. Also surprisingly, ConA lectin binding, which is specific to Manα2/6/3-manβ4-Glc(Nac)-R > α-Man > α-Glc > αGlcNAc motifs, was inhibited after the action of two clones (40B3 and 12O6) which were not detected as being active on X-α-Man. We cannot exclude the possibility that these clones do indeed produce the enzymes required to break down the glycan motifs targeted by ConA, but that their activity cannot be detected on the corresponding X-glycosyl substrates due to the structural modification of the aglycon in chromogenic substrates. Another explanation could be the production by the hit clones of cell wall anchors or glycoprotein adhesins (as further explained in this paper regarding 9D11 sequence annotation), which may compete for binding with mucin-specific lectins.

### Identification of Mucin Glycan Utilization Loci

The metagenomic DNA from the 13 clones was sequenced using a high sequencing depth (100X) to ensure reliable sequence assembly. For each clone, one contig of between 27,897 and 50,904 bp in size was obtained. In total, 794,676 bp (containing 316 ORFs) were analyzed. Of these 316 ORFs, 56 were annotated as CAZyme encoding genes.

Forty-nine of these predicted CAZymes contained a GH module (Supplementary Table S1), and a further four were esterases. In most of the contig sequences, the CAZyme-encoding genes were organized in operon-like multigenic systems similar to the polysaccharide utilization loci (PULs) described for cultivated species (Terrapon et al., 2018) and other metagnomic DNA fragments (Tasse et al., 2010; Supplementary Table S1). The metagenomic PULs retrieved in the present study code for the batteries of CAZymes, carbohydrate sensing/binding proteins and transporters required to achieve the complete breakdown and uptake of mucin glycans. The structural modification of mucin glycans results from the production of several glycosidase activities that could be inferred from the GH families highlighted in their sequences (Table 2 and Supplementary Table S1), and which are all required for the degradation of complex mucin glycans. Most of the GH sequences presented the signal peptide sequences, indicating their membrane or extracellular location in the bacteria they originate from, necessary to initiate the breakdown of complex glycan structures into simpler oligosaccharides, which are very likely internalized thanks to the various carbohydrate transporters we identified (annotated as ABC transport family, MFS, SusD/SusC systems or outer membrane efflux protein). As with canonical Bacteroidetes PULs, we also identified other genes coding for proteins involved in the regulation of gene expression in these metagenomic loci (AraC family transcriptional regulator, chemotaxis protein CheY/response regulator receiver domain protein). Finally, as described in greater detail later in this section, we also identified in these loci several genes coding for putative enzymes involved in central carbohydrate metabolism, especially sialic acid metabolism.

**FIGURE 2:**
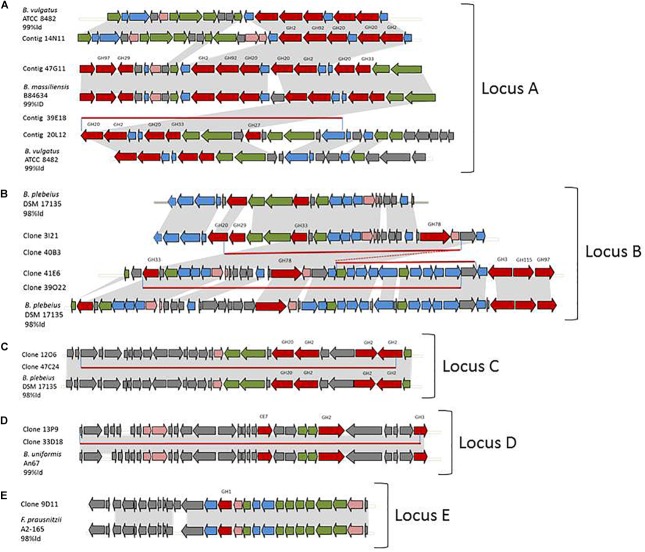
Metagenomic loci of the hit clones and syntenies with reference genomes. **(A)** partial redundancy between clones 14N11, 47G11, 39E18, and 20L12 and their synteny with *B. vulgatus* ATCC 8482 and *B. massiliensis* B84634. **(B)** partial redundancy between clones 3I21, 40B3, 41E6, and 39O22 and their synteny with *B. plebeius* DSM 17135. **(C)** partial redundancy between clones 12O6 and 47C24 and their synteny with *B. plebeius* DSM 17135. **(D)** partial redundancy between clones 13P9 and 33D18 and their synteny with *B. uniformis* An67. **(E)** synteny of 9D11 and *F. prausnitzii* A2-165. In gray, identical genome parts; red arrows, CAZymes; blue arrows, other enzymes of carbohydrate metabolism; green arrows, transporters; pink arrows, transcription/transduction signal; gray arrows, others; and red lines, redundant metagenomic sequences.

Partial or complete sequence redundancy was observed in clones 14N11, 20L12, 47G11, and 39E18, in clones 3I21, 40B3 41E6, and 39O22, in clones 12O6 and 47C24, and in clones 13P9 and 33D18 (Figure 2A–D, respectively). These 12 sequences finally correspond to four loci, all assigned to the *Bacteroides* genus using PhyloPythiaS which is in agreement with the syntenies detailed hereafter.

### Locus A Assigned to *Bacteroides vulgatus* or *Bacteroides massiliensis*

The three partially redundant sequences of the sialidase-, β-D-NAcetylgalactosaminidase- and/or β-D-NAcetylglucosaminidase-producing clones 14N11, 20L12, and 39E18 presented high sequence identity (99–100%) onto their coverage areas (Figure 2A). The 39E18 contig is included in that of 20L12. The 14N11 and 20L12 sequences can be combined to form a longer, single contig containing eight GH-encoding genes assigned to the GH2 (2 sequences), GH20 (3 sequences), GH27, GH33, and GH92 families. These families all host already characterized members which are able to depolymerize human N- and O-glycans; β-galactosidases, β-N-acetylglucosaminidases, α-N-acetylgalactosaminidases, sialidases and α-mannosidases, respectively. Synteny was detected between contigs 14N11, 20L12, and 39E18 and a locus from *Bacteroides vulgatus* ATCC 8482 corresponding to the predicted PUL 67. The synteny with PUL 67 is, however, disrupted by the insertion of four genes in the metagenomic DNA (ORFs 13–16 in clone 20L12), including the GH27 encoding gene and two genes involved in nutrient binding and transport. This insertion confers an ecological advantage to the uncultured strain containing this metagenomic locus over *B. vulgatus* for O-GalNAc glycan foraging, especially the core 3 structure from human colonic mucins (Brockhausen et al., 2009). Sialidase activity was observed for contig 47G11 on the chromogenic substrate and mucus. This contig contained a similar gene series to 14N11 and 20L12, albeit with two additional CAZyme-encoding genes (GH29 and 97), and shared, respectively 82 and 90% sequence identity with contigs 14N11 and 20L12. The percentage of its CAZyme sequences’ identity with the homologous sequences in contigs 14N11 and 20L12 (79–90%) indicates that these metagenomic fragments originate from different strains or species. Contig 47G11 indeed presents an almost perfect synteny with the predicted PUL 4 from *B. massiliensis* B84634 (100% Cov, 99% ID), but with two additional genes in the metagenomic sequence.

**FIGURE 3:**
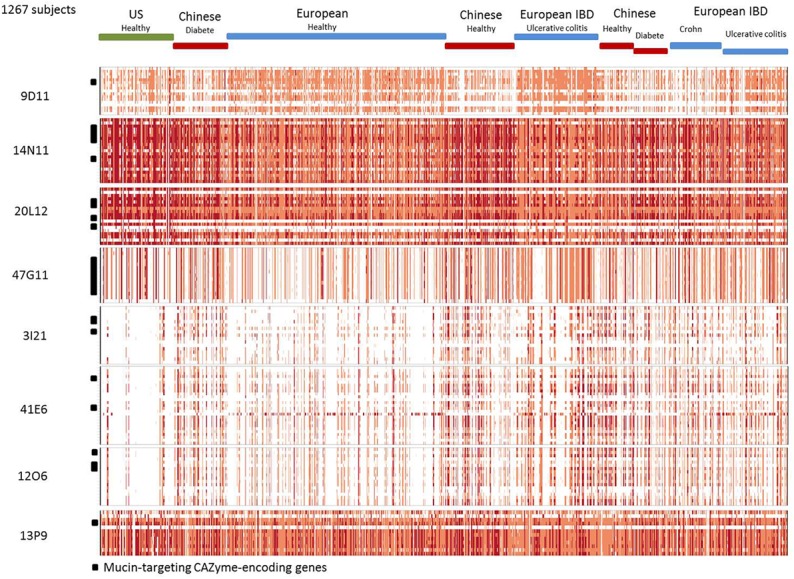
Abundance and prevalence of the genes of the contigs in the human gut metagenome. Genes are in rows. Individuals are in columns. The normalized abundance is represented by a color scale, white, not detected; pink, orange, and red, increasing abundance with a 100-fold change between colors.

The *B. vulgatus* strain ATCC 8482 is one of the most prevalent strains in the human gut *Bacteroides* group. It is thought to be responsible for the development of inflammation and exacerbated immune response in the pathogenesis of IBD (Shiba et al., 2003). However, in our study, we found the genes of contigs 20L12 and 14N11 to be highly prevalent and abundant in the human gut metagenomic gene catalog, regardless of the geographic origin or medical status of the subjects (Figure 3 and Supplementary Table S1). The locus identified in this study, which is not a biomarker of IBD, is thus unlikely to be involved in IBD. Besides, the *B. vulgatus* strain ATCC 8482, which is known to produce a whole panel of glycolytic activities required to degrade mucus glycoproteins (Ruseler-van Embden et al., 1989), has previously been found to degrade pig gastric mucin, but not human mucin glycans (Hoskins et al., 1992; Png et al., 2010). These data could thus be revisited by more specifically assessing the growth of this strain on specific structures of sialylated mucin glycans. As *B. vulgatus* strain ATCC8482, *B. massiliensis* B84634 was also reported to be able to grow on pig gastric mucin, resulting in the activation of six of its 33 PULs, including PUL 4 (Pudlo et al., 2015). The prevalence of the cluster’s genes is lower than those of corresponding genes in the *B. vulgatus* strain, and their abundance is lower in European subjects (Figure 3 and Supplementary Table S1).

Finally, in the contigs of these sialidase-producing clones, we also identified genes encoding putative sialic acid-specific 9-O-acetylesterase, N-acetylglucosamine-6-phosphate deacetylase, glucosamine-6-phosphate deaminase and sialic acid-induced mutarotase, constituting the denominated Nan system for *N*-acylneuraminate (Vimr et al., 2004; Egan et al., 2014). Marcobal et al. (2011) stressed that a certain number of bacterial species do not possess all the enzymes for the complete catabolism of sialic acid: *B. tetaiotaomicron*, for example, has sialidases but not the Nan system to metabolize sialic acid. Conversely, other species such as *Clostridium difficile* do not have sialidases but possess the Nan system to metabolize sialic acid released by other bacteria. Here, the uncultured bacteria from which contigs 14N11, 20L12, and 39E18 originated evolved to cluster all the sialidase-encoding genes and the Nan operon on the same locus, as well as other CAZyme-encoding genes likely to release other monosaccharides belonging to the sialylated mucin glycans. This confers an important ecological advantage to colonize the mucosal layer.

### Locus B Assigned to *Bacteroides plebeius*

The four partially redundant sequences 3I21, 40B3, 39O22, and 41E6 have high sequence similarity (99–100%) (Figure 2B). The 3I21 and 41E6 sequences can be combined to form a longer, single contig containing seven GH-encoding genes, including the GH20, GH29, GH33, GH3 families, which are known to contain members able to depolymerize O-glycans (β-1,6-N-acetylglucosaminidases, α-L-fucosidases, sialidases). The remaining GH78-, GH115-, and GH97-encoding genes belong to another gene cluster best suited to the degradation of plant glycans, which highlights the probable metabolic flexibility of the uncultured bacterium from which this metagenomic DNA fragment originated. The functional profile of the clones matches their content in GH families (Table 2 and Figure 1). Additional genes involved in carbohydrate binding and transport (annotated as substrate-binding protein, SusC/SusD, and major facilitator family transporter) and gene expression regulation were also present in these metagenomic sequences. These contigs present a nearly perfect synteny with the predicted PUL 9 from the *B. plebeius* DSM 17135 strain (93% Cov, 98% Id for 3I21 and 95% Cov, 98% Id for 41E6), although this was disrupted by the insertion of two genes coding for a GH29 putative α-L-fucosidase (ORF6 in contig 3I21) and one putative transporter (ORF18 in contig 3I21), adding a complementary function to the bacterium’s glycan degradation pathway. In this mucin-targeting metagenomic PUL, GH-encoding genes were also surrounded by genes conferring complementary functions for N-acetylhexosamine catabolism and sialylation processes – methyl transferase, N-acetylneuraminate cytidylyltransferase, N-acetylneuraminic mutarotase, sialic acid-specific 9-O-acetylesterase, N-acetylneuraminate lyase and N-acylglucosamine 2-epimerase – whose roles in carbohydrate metabolism have previously been described for symbiotic and pathogenic bacteria (Vimr and Lichtensteiger, 2002; Ringenberg et al., 2003; Bravo et al., 2004; Vimr et al., 2004; Mizanur and Pohl, 2008). This gene cluster thus combines genes dedicated to the depolymerization of sialylated mucin glycans with genes involved in the synthesis, activation and transfer of sialic acid onto bacterial cell surfaces. Such a process of carbohydrate harvesting and reuse for bacterial glycan synthesis would permit bacteria to mimic vertebrate cell surfaces and evade the host’s immune system. In mammals, sialic acids are widespread molecules usually at the terminal position of oligosaccharide chains of cell-surface or serum glycoconjugates. Their functions in regulation of host innate defense mechanisms make it a key substance for microorganisms to survive in the environment (Vimr et al., 2004). Although they were all prevalent in the sample, these metagenomic genes are not evenly distributed among the population (Figure 3). Except for one gene (ORF1, contig 41E6), a mobile element appearing in the heat map as a biomarker of European subjects, the entire locus (including the additional GH29 compared to *B. plebeius* DSM 17135 PUL9) is significantly more abundant in the European IBD cohort than the healthy cohort. At the same time, it is abundant and prevalent in the Chinese subjects, including the healthy ones, and almost absent in the United States subjects.

### Locus C Assigned to *Bacteroides plebeius*

Contig 47C24 is included in contig 12O6, with which it shares 99% sequence identity. Contig 12O6 has 98% identity with a *B. plebeius* DSM 17135 locus, which includes a part of the predicted PUL 4 (Figure 2C). This part of PUL 4 contains one GH20- and three GH2-encoding genes, with the former gene explaining the β-D-Nacetylgalactosaminidase and β-D-Nacetylglucosaminidase activities of clones 47C24 and 12O6. Surrounding the CAZymes, we also found three genes likely involved in the binding and transport of nutrients. Finally, not surprisingly, the prevalence and abundance pattern of this locus was similar to that of contigs 3I21, 40B3, 39O22, and 41E6, which also showed synteny with other *B. plebeius* DSM 17135 loci.

### Locus D Assigned to *Bacteroides uniformis*

The last *Bacteroides* contig, namely contig 13P9, which includes sequence 33D18 (Figure 2D), shared 99% identity with a genomic locus from *B. uniformis* An67, a strain which is not included in the PUL database. This metagenomic PUL contains a GH2-, a GH3-, and a CE7-encoding gene. The GH2 family contains β-D-mannosidases, explaining activity on X-β-D-Man (Table 2 and Figure 1). Due to its position at the end of the contig, the GH3-encoding gene is truncated and probably not functional here, but some of the characterized proteins of the family are known to act on β-N-acetylhexosamines, of which the acetyl groups could be hydrolysed by the CE7 enzyme. This metagenomic locus is probably involved in the catabolism of human N-glycans by uncultured bacteria, and/or *B. uniformis* strains, which have a high potential to utilize both dietary (Tasse et al., 2010; Patrascu et al., 2017) and endogenous glycans (Benítez-Páez et al., 2017). This considerable metabolic flexibility of *B. uniformis*, which gives it a significant advantage when it comes to easily colonizing the human gut ecosystem, could explain why the locus identified in this study is so prevalent and abundant in the microbiome, regardless of the geographic origin or medical status of the individual (Supplementary Table S1 and Figure 3).

### Locus E Assigned to *Faecalibacterium prausnitzii*

The last metagenomic locus identified here, that of the β-D-mannosidase and β-N-acetylglucosaminidase clone 9D11, was assigned to Feacalibacterium using PhylophythiaS. It was indeed found to share 98% identity with a locus of *Faecalibacterium prausnitzii* A2-165 F (Figure 2E). It contains only one CAZyme-encoding gene (GH1), which was probably responsible for the activity detected on X-β-Man. Conversely, neither activity on X-β-GlcNac nor modification of WGA and SNA lectin binding to mucus could be explained by the presence of this GH1 enzyme, indicating that one or several of the numerous putative proteins encoded in this locus (some presenting distant homologies with adhesins and agglutinins) may perform these functions, and/or that the encoded GH1 is a highly promiscuous enzyme, which will have to be characterized. Additionally, five genes coding for a putative peptidic transport system (oppABCDF operon) were clustered in this locus. The opp transport system is involved in peptide uptake for nutrition, sensing environmental changes and recycling peptides from, for instance, muropeptides; that is, peptidoglycans from bacterial cell walls consisting of alternating residues of β-N-acetylglucosamine (NAG) and N-acetylmuramic acid (NAM) linked to a peptide chain (Monnet, 2003). In this pathway, peptides internalized by the oppABCDF system are hydrolysed into amino acids by a set of peptidases, a role that may have been performed here by the putative CocE/NonD hydrolase belonging to the aminopeptidase family identified in the 9D11 locus. With respect to the functioning of *F. prausnitzii* in the human gut microbiota, most strains grew on N-acetylglucosamine, highlighting their probable ability to utilize host-derived substrates (Lopez-Siles et al., 2012). This is consistent with the CAZy gene content of the *F. prausnitzii* genomes. We indeed found several *F. prausnitzii* sequences in the CAZy database that had been assigned to families as GH2- and GH3-containing members active on β-D-N-acetylglucosamine, β-D-N-acetylhexosamine and β-D-glucosamine linkages, although none of these *F. prausnitzii* enzymes has been biochemically characterized to date. In any case, *F. prausnitzii* is far from being as well equipped with glycan-degrading enzymes as the *Bacteroides* species described above. Nevertheless, our results demonstrate that this species has the potential to interact with mucus. This could explain the above mentioned modulation of the effect of *B. thetaiotaomicron* on the intestinal mucus barrier, resulting in a modification in goblet cell differentiation and mucin glycosylation (Wrzosek et al., 2013). Moreover, *F. prausnitzii* belongs to the most abundant Firmicutes species in the human gut, which is consistent with the high abundance and prevalence of the locus we identified in this study (Figure 3). The gene abundance heat map indicates a lower prevalence of 9D11 genes in Chinese subjects, regardless of their medical status, than in European ones. What is more, compared to healthy subjects, these genes are more abundant in the microbiome of patients suffering from ulcerative colitis, but less abundant in patients with Crohn’s disease (CD). This is perfectly consistent with the low abundance of *F. prausnitzii* in the microbiome of CD patients, particularly those with ileal involvement (Sokol et al., 2009), which could be connected with the ability of this species to interact with mucus through its glycan and proteic fractions, as highlighted here with the 9D11 locus.

## Conclusion

To conclude, our work constitutes the first activity-based metagenomic study targeting the glycan-mediated relationships between the human gut microbiota and the host. Until now, the identification of bacterial species and metabolic pathways involved in mucus degradation was restricted to cultured bacteria, tested for growth on complex mucin glycan structures of porcine origin. When combined to genomic and transcriptomic analyses, these studies allowed to identify mucin glycan utilization loci for the few dozen of targeted strains. Nevertheless, to date only a very small number of these pathways have been biochemically proven to degrade human mucin structures.

In the present study, we designed a two-step functional screening strategy, which allowed us, for the first time, both to identify mucus degrading pathways from uncultured species of the microbiota, and to prove their ability to degrade specific motives of human colonic mucus. The mucin glycan utilization loci we discovered are highly prevalent and abundant in the microbiome, and present marked synthenies with loci from several prominent commensal strains from the Bacteroidetes and Firmicutes phyla, most of them being known to forage on dietary glycans, but which had never been described to feed on mucin glycans. Our data highlight the metabolic flexibility of these abundant commensals, which would have the ability to easily redirect their metabolism from dietary polysaccharides to host-derived glycans, depending on the availability of resources. In addition, we highlighted the overabundance of certain of these (meta) genomic loci in the microbiome of patients suffering from inflammatory diseases. This particular trait, together with the proof of their involvement in the modification of human mucus, makes them new targets to study the host response to the degradation of the mucus firewall, and to elaborate new strategies to restore gut homeostasis, for example by using specific functional foods to divert over-abundant mucus foragers toward dietary glycans.

## Ethics Statement

Bacterial metagenomic DNA was obtained from the distal ileum sample of a 51-year-old male patient undergoing colonoscopy and surgery for a suspected cancer of the lower colon after he had been subjected to a cleansing preparation. The patient did not receive any antibiotics or other drugs in the 6 months before sampling. The sampling protocol was approved by the local ethics committee: the Comité de Protection des Personnes Sud Est V (Ref.: 07-CHUG-21; B. Habozit, J.L. Crakowski, J. Juge, J. Grunwald, E. Svhan, and E. Fontaine on the committee) and informed written consent was obtained from the subject before sampling. The human colon mucus was isolated from patients (Ajandouz et al., 2016). Briefly, human intestinal colonic resections (5–10 cm long) were obtained from patients undergoing surgery for colonic adenocarcinoma at the Hospital Nord of Marseille’s general and gastrointestinal surgery unit as part of a collaborative “clinical transfer” project. The sampling procedures were approved by the French Ethics Committee (CODECOH no. DC-2011-1319).

## Author Contributions

EL conceived the study, designed the work, acquired, analyzed and interpreted the data, and drafted the work. JP and MM conceived the study, designed the work, acquired and analyzed the data, and drafted the final revision of the manuscript. NB and JE acquired, analyzed and interpreted the data, and drafted the final revision of the manuscript. AT, EB, ED, and BH analyzed and interpreted the data, and drafted the final revision of the manuscript. ML, SB, EDP, and PR conceived the study and designed the work, and drafted the final revision of the manuscript. GP-V conceived the study, designed the work, analyzed and interpreted the data, and drafted the final revision of the manuscript.

## Conflict of Interest Statement

PR was employed by company Givaudan SA. The remaining authors declare that the research was conducted in the absence of any commercial or financial relationships that could be construed as a potential conflict of interest.

## Footnotes

1 https://www.carbosynth.com

2 http://www.genome.umd.edu/masurca.html

3 http://bozeman.mbt.washington.edu/phredphrapconsed.html

4 http://rast.nmpdr.org/

5 http://www.cazy.org

6 http://phylopythias.bifo.helmholtz-hzi.de

7 http://www.cbs.dtu.dk/services/SignalP/

8 http://meta.genomics.cn/meta/dataTools

9 https://www.ncbi.nlm.nih.gov/genbank/
